# Comparison of radiographic scoring systems for assessment of bone healing after tibial plateau leveling osteotomy in dogs

**DOI:** 10.3389/fvets.2023.1147386

**Published:** 2023-04-06

**Authors:** R. A. Leal, N. E. Lambrechts, J. D. Crowley, J. F. Griffin, J. J. Karnia, B. T. Torres, K. C. Maritato, N. R. Kieves, F. M. Duerr

**Affiliations:** ^1^College of Veterinary Medicine and Biomedical Sciences, Colorado State University, Fort Collins, CO, United States; ^2^Small Animal Specialist Hospital, North Ryde, NSW, Australia; ^3^Department of Large Animal Clinical Sciences, Texas A&M University, College Station, TX, United States; ^4^College of Veterinary Medicine, University of Missouri, Columbia, MO, United States; ^5^MedVet Medical and Cancer Center for Pets, Cincinnati, OH, United States; ^6^College of Veterinary Medicine, Ohio State University, Columbus, OH, United States

**Keywords:** tibial plateau leveling osteotomy (TPLO), osteotomy healing, radiograph (x-ray), osteotomy, bone healing

## Abstract

**Introduction:**

Accurate radiographic assessment of bone healing is vital in determining both clinical treatment and for assessing interventions aimed at the promotion of bone healing. Several scoring systems have been used to evaluate osteotomy changes following tibial plateau leveling osteotomy (TPLO). The goal of this study was to compare the ability of five radiographic scoring systems to identify changes in bone healing following TPLO over time (Aim I), and to evaluate the influence of limb positioning on TPLO osteotomy scoring (Aim II).

**Materials and methods:**

Phase I-A randomized, blinded, prospective study was conducted using similarly positioned postoperative TPLO radiographs from seven dogs taken immediately postoperatively, 6-weeks, and 8-weeks postoperatively. Ten reviewers assessed the radiographs, and five different scoring systems were tested for each set including three previously published ones, a Visual Analog Score (VAS), and a subjective 11-point scale. For each system, responses for 6-week postoperative were compared to 8-week postoperative. Scores were judged as correct (=showing an increase in score), incorrect (=decrease in score), or unchanged (=same score). Phase II-An international group of 39 reviewers was asked to score radiographs from three dogs, taken in different positions, using the VAS grading system. Scores were averaged and comparisons were made for each set.

**Results:**

Phase I-The VAS system identified the greatest number of sets correctly (76%), with the least unchanged scores (15%), and 9% incorrect scores. Phase II-All three patients had an increase in the average difference between VAS-scores for differently positioned radiographs compared to similarly positioned radiographs. The magnitude of change between different positions far exceeded the magnitude of comparison of the similarly positioned radiographs from the 6- and 8-week time point.

**Discussion/Conclusion:**

The VAS system appears to be the most appropriate of the tested systems to identify small changes in bone healing. In addition, the positioning of postoperative TPLO radiographs makes a substantial difference in the healing score that is assigned. Care must be undertaken when performing postoperative radiographs in both the clinical and research setting to ensure accurate assessment of bone healing.

## Introduction

Cranial Cruciate Ligament Disease (CCLD) is a common cause of pelvic limb lameness in dogs (1). The disease is frequently managed with tibial plateau leveling osteotomy (TPLO) to dynamically stabilize the cranial cruciate ligament deficient stifle (2). Most TPLO osteotomies are considered healed sufficiently to start the return to normal activity between 6 and 12 weeks with an average of ~8 weeks (3, 4). Postoperative return to increased activity is frequently based on radiographic evidence of healing of the osteotomy, in combination with physical examination findings (5, 6), although the need to perform recheck radiographs to confirm osteotomy healing in dogs recovering normally has recently been questioned (7).

As a common and fairly standardized osteotomy with internal fixation for management of CCLD, TPLO patients may pose a useful clinical model to investigate variables that affect osteotomy healing (1, 8, 9). Various methods that evaluate radiographic TPLO osteotomy healing have been described, including different variations of 5-, 10-, and 12-point scales, however, there is no agreed upon grading standard (8–11). With many different grading scales, there are bound to be variations in degree of healing reported due to the grading methodologies. The use of different scales makes it difficult to compare the effect of different treatments between studies. In addition, because of the unique features of the TPLO osteotomy, namely the circular osteotomy, metaphyseal location, and use of a custom-shaped bone plate, positioning of the limb is likely to have a substantial impact on radiographic appearance. However, it is currently unknown whether positioning differences create clinically relevant changes in osteotomy scoring.

Accuracy (i.e., true degree of bone healing) of any TPLO scoring system that relies on radiographic assessment is difficult to determine. In addition to a thick metal plate obstructing the view of the osteotomy line, radiography is insensitive at determining the degree of mineralization of osteoid and is a poor measure of bone stiffness and therefore bone healing (12–14). In fact, the osteotomy line may be completely filled at the 6–8 week healing mark, but with structurally weak lamellar bone (15). In human medicine, this limitation of orthogonal radiographs is addressed by evaluating fracture healing *via* a combination of imaging (radiography or CT), clinical assessments, and patient reported metrics (16). New methods for evaluation of fracture healing are being explored, such as contrast-enhanced ultrasonography to measure neovascularization of a fracture site, but their use is not wide-spread to date (17, 18). In the veterinary profession, radiography is the most commonly used method of evaluating bone healing (19, 20). This use is most likely due to equipment availability, relative low cost, and familiarity of interpretation when compared to the other modalities.

Identification and establishment of a TPLO osteotomy scoring system that best utilizes this imaging modality is vital to future research on bone healing. Therefore, the objective of this study was to compare the ability of five different radiographic scoring systems to identify subtle changes in bone healing after TPLO in dogs. The second objective was to assess the influence of limb positioning on radiographic healing after TPLO.

## Methods

### Study design

A randomized, blinded, prospective study (phase I) was conducted using postoperative TPLO radiographs from dogs that underwent TPLO and had participated in an unrelated clinical trial (CSU VTH CRB #2018-177). These radiographs contained standard orthogonal views taken immediately postoperatively, at ~6-, and 8-weeks postoperatively. Only radiographs from patients that healed without complication, did not have additional procedures (e.g., patellar luxation repair) performed, and where positioning of the limb was similar at all time points were selected for the study.

Following identification of a reliable grading system derived from phase I, a second randomized, blinded, prospective study (phase II) was conducted using additional postoperative TPLO radiographs from the same clinical trial. These radiographs contained standard orthogonal views taken immediately postoperatively and at ~6-, and 8-weeks postoperatively. At the 6- and 8-week time-points, slight variations of the standard “TPLO-views” were obtained in addition to the standard views (21). The same healing and procedural criteria as above applied.

### Phase I: Scoring system identification

#### Radiographic views

Each set of orthogonal radiographs included the distal femur from proximal to the femoro-patellar joint and the entire tibia and tarsocrural joint performed as standard “TPLO-views” (21). Additionally, each radiograph was reviewed at the time of postoperative radiograph acquisition to confirm that the craniocaudal view showed the limb positioned such that the entire medial cortex of the proximal tibia was visualized and was not obscured by the plate. The mediolateral view was obtained aiming to position the limb such that the beam was parallel with the orientation of the osteotomy site. Positioning specifics (e.g., amount of external or internal rotation of the tibia and elevation of the lower limb) were recorded and pictures were obtained to accomplish the same positioning at the time of recheck radiographs. Radiographs were edited to eliminate dead space and included only the distal femur from proximal to the femoro-patellar joint and the proximal half of the tibia. Additional radiographic sets were “fabricated,” by rotation or enlargement of some of the images above to give the appearance of a different image.

#### Radiographic scoring sessions

**Figure 1:**
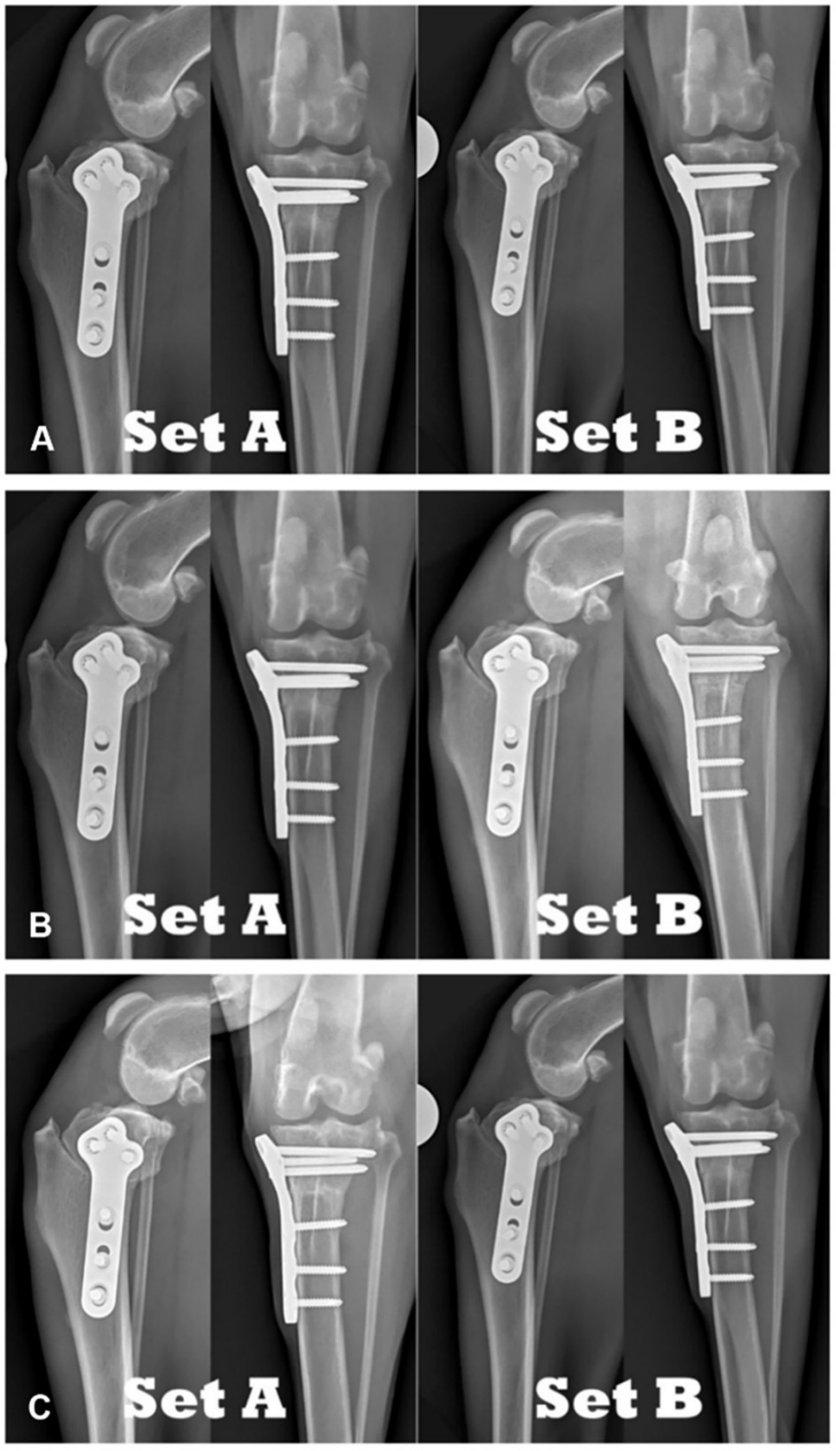
Comparison of TPLO radiographs of the same patient: **(A)** 8-weeks postoperative compared to 8-weeks postoperative. **(B)** 8-weeks postoperative compared to 6-weeks postoperative. **(C)** 6-weeks postoperative compared to 8-weeks postoperative.

**Figure 2:**
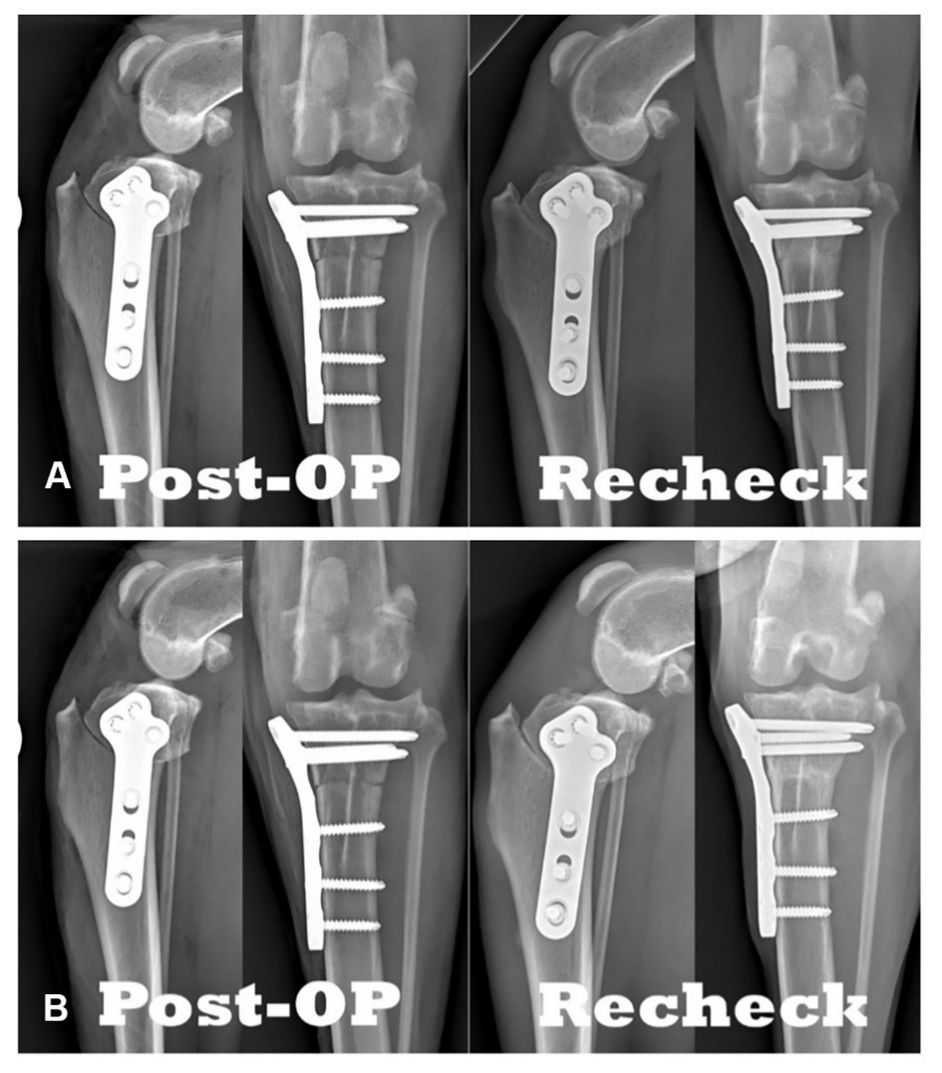
Comparison of TPLO radiographs: **(A)** Immediately postoperative compared to 8-weeks postoperative. **(B)** Immediately postoperative compared to 6-weeks postoperative.

Reviewers were asked to evaluate 20 total sets of radiographic comparisons of two orthogonal views of the stifle joint (i.e., 4 radiographs per set) from four patients using five scoring systems. Each set (see Figure 1) was clearly marked, assigned a scoring system if indicated, and randomized. These sets belonged to one of 5 categories. Three of these categories (12 total sets from 4 patients) showed a comparison of recheck radiographs and two of these categories (8 total sets from 4 patients) showed a comparison between the postoperative radiographs and a recheck radiograph: (1) comparison of 6- vs. 8-weeks (i.e., set A = 6 weeks and set B = 8 weeks), (2) comparison of 8- vs. 6-weeks (i.e., set A = 8-weeks and set B = 6-weeks), (3) comparison of 8- vs. 8-weeks (i.e., the same sets of 8-week postoperative radiographs provided twice), (4) immediate postoperative with 6-weeks (i.e., set A = postoperative and set B = 6-weeks), (5) immediate postoperative with 8-weeks (i.e., set A = postoperative and set B = 8-weeks); see Figures 1, 2 for examples. The first three categories were provided to confirm that, when directly compared, reviewers were able to identify a difference in bone healing between the two time points (i.e., to confirm an actual detectable change across the 2-week time frame). Categories 4 and 5 were evaluated using each of the randomly assigned scoring systems during each review session. Both the craniocaudal and mediolateral projections were utilized in the scoring of the radiographs. Reviewers were asked to assess bone healing during 3 separate sessions with a minimum of 2 weeks between sessions to avoid recall bias. Evaluators were blinded by removing all identifying features and labels from the radiographs and labeling them with a random number and letter combination (A-Z and 1–1000).

#### Radiographic scoring systems

For the radiographic sets comparing recheck radiographs (see Figure 1), reviewers were asked to determine which set appeared more healed (i.e., represents the 8-week timepoint) or whether they were from the same timepoint (i.e., two sets of radiographs representing 8-week timepoint).

For comparison of postoperative radiographs to recheck radiographs (see Figure 2), the following five scoring systems were utilized:

##### Scoring system #1

Subjective 5 score (SUB5)–This previously described scoring system utilizes a five-point scale (9, 22, 23). The reviewers were asked to provide their subjective assessment of osteotomy healing (i.e., bony bridging, callus formation, osseous remodeling etc.) of the recheck radiographs based on both radiographic projections. The scores were defined as: 0 = no healing, 1 = 1–25% healed, 2 = 26–50% healed, 3 = 51–75% healed, 4 = 76–100% healed.

##### Scoring system #2

Subjective 11 score (SUB11)-This novel scoring system utilizes an 11-point scale. The same instructions as for SUB5 were provided, however, reviewers were asked to score on an expanded scale: 0 = no healing, 1 = 1–10% healed, 2 = 11–20% healed, 3 = 21–30% healed, 4 = 31–40% healed, 5 = 41–50% healed, 6 = 51–60% healed, 7 = 61–70% healed, 8 = 71–80% healed, 9 = 81–90% healed, 10 = 91–100% healed.

##### Score system #3

**Figure 3:**
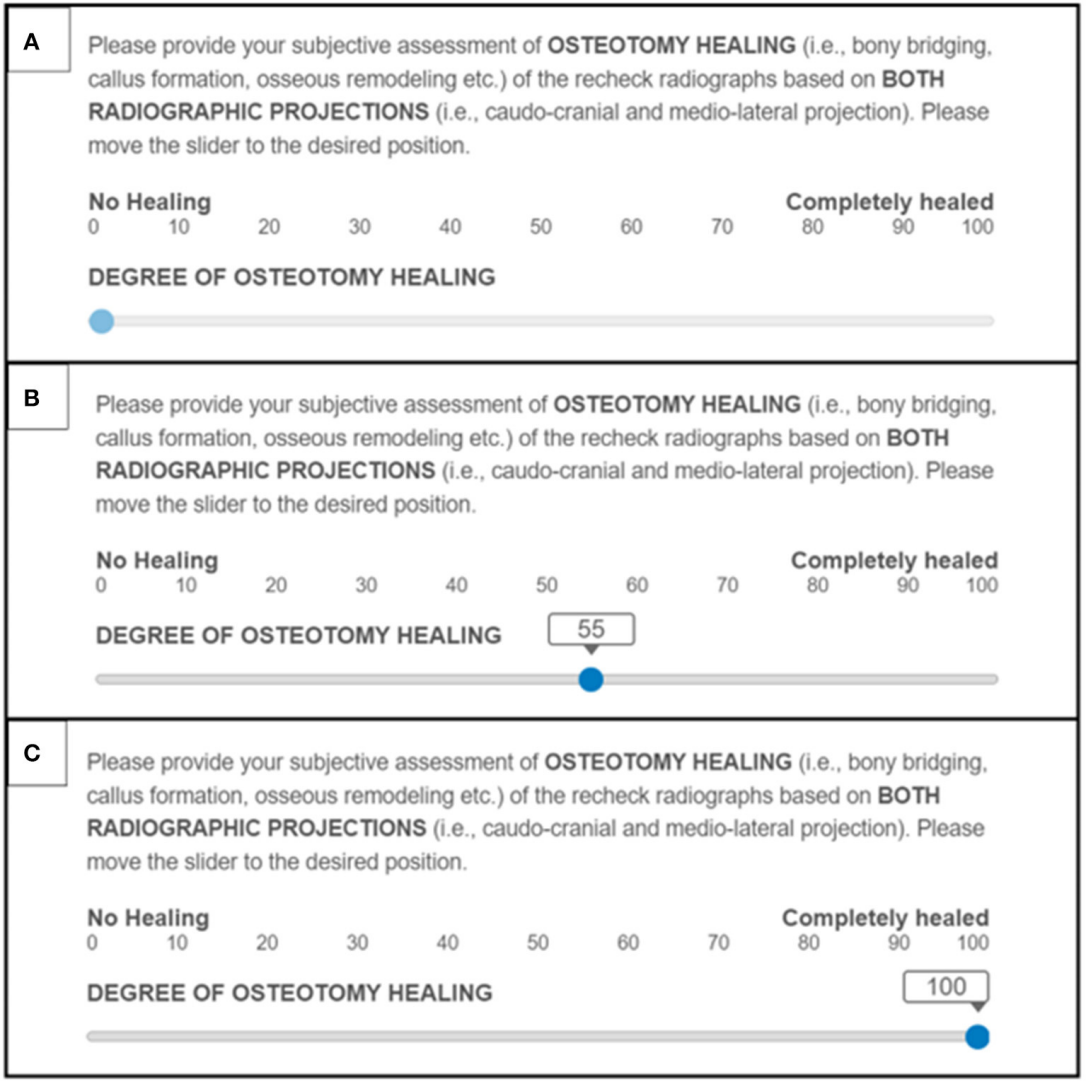
Examples of the Visual Analog assessment score system used to grade TPLO osteotomy healing: **(A)** Slider placement representing 0% healed. **(B)** Slider placement representing 55% healed. **(C)** Slider placement representing 100% healed.

Visual Analog score (VAS)–This system is a novel variation of the SUB5 scale (9). For this system, the same instructions as for SUB5 were provided however, reviewers were asked to move a virtual slider along a 100-point scale. The scale was labeled at either end: 0 with “no healing” and 100 with “completely healed” (see Figure 3).

##### Scoring system #4

Descriptor score (DESCRIPTOR)–This previously described scoring system utilizes a combination of cortex healing descriptors and osteotomy healing descriptors to provide a healing score of 0–4 (24). The reviewers were asked to provide an assessment of osteotomy healing of the recheck radiographs based on both radiographic projections using the following scale: 0 = easily seen osteotomy without any evidence of closure and distinct margins, 1 = easily seen osteotomy, but margins indistinct, 2 = moderately difficult to see the osteotomy margins, 3 = difficult to see the osteotomy margins, 4 = osteotomy margins are not seen at all.

##### Scoring system #5

Bridging score (BRIDGING)–This previously described scoring system utilizes a simple yes or no evaluation (25). The reviewers were asked to provide their subjective assessment of osteotomy healing, specifically whether there was complete iso-opaque bridging of 2 or more cortices present in the recheck radiographs.

### Phase II: Effect of positioning

#### Radiographic views

**Figure 4:**
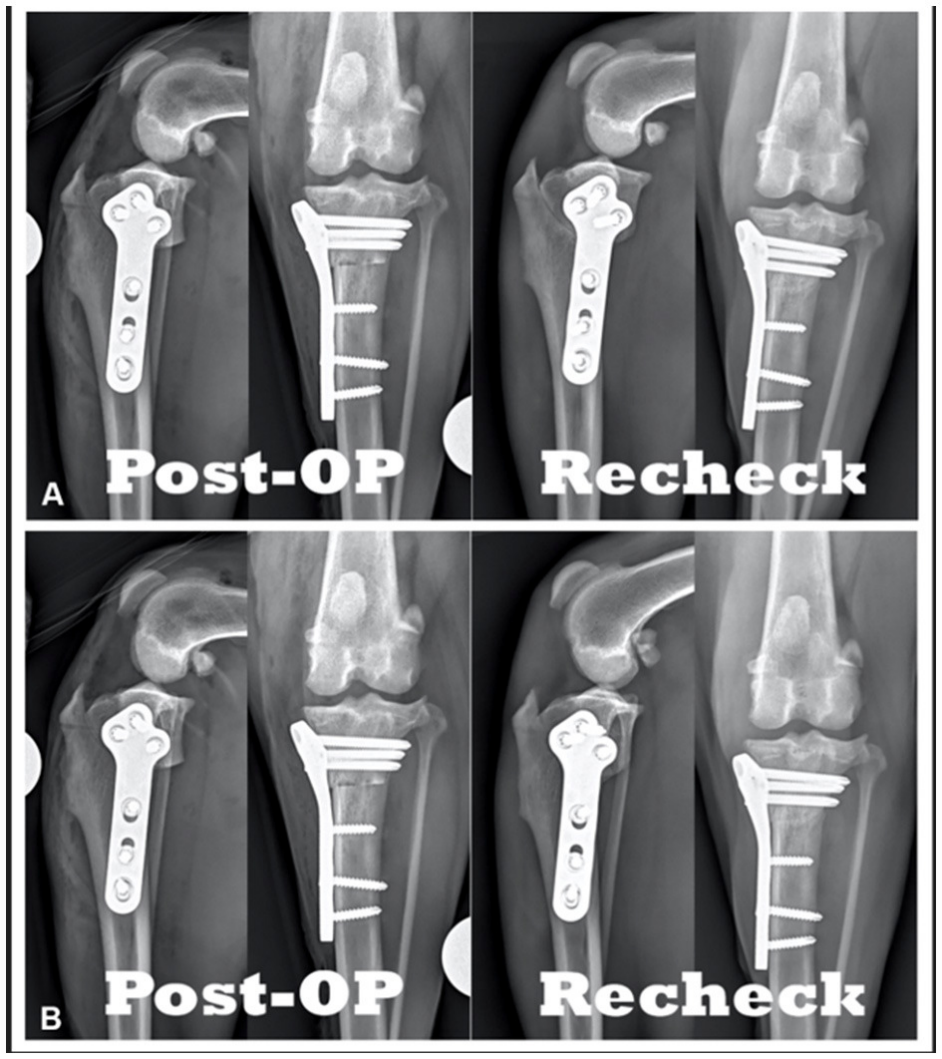
Comparison of TPLO radiographs of the same patient: **(A)** Immediately postoperative vs. 6-weeks postoperative radiographs. **(B)** Immediately postoperative vs. 6-weeks postoperative with slight variation in positioning.

As the radiographs were obtained during phase I, the following additional views were obtained. At each time-point and for both the mediolateral and craniocaudal views, additional radiographs were taken as standard “TPLO-views” but with a slight rotation to the limb (see Figure 4). One dog had these additional views performed at 6-week postoperative while the remainder of the dogs in the study had these additional views performed only at 8-weeks postoperative.

#### Radiographic scoring sessions

Reviewers were asked to evaluate 17 total sets of comparisons of two orthogonal views of the stifle joint (i.e., 4 radiographs per set) from five patients. Each set was clearly marked and randomized. These sets belonged to one of five categories. The first three of these categories (2 patients with 3 sets each) showed a comparison of recheck radiographs and the last two of these categories (2 patients with 3 sets each, 1 patient with 5 sets) showed a comparison of the postoperative radiographs to a recheck radiograph: (1) comparison of 6- vs. 8-weeks (i.e., set A = 6 weeks and set B = 8 weeks), (2) comparison of 8- vs. 6-weeks (i.e., set A = 8-weeks and set B = 6-weeks), (3) comparison of 8- vs. 8-weeks (i.e., the same sets of 8-week postoperative radiographs provided twice), (4) immediate postoperative with 6-weeks (i.e., set A = postoperative and set B = 6-weeks), (5) immediate postoperative with 8-weeks (i.e., set A = postoperative and set B = 8-weeks). The first three categories were similarly provided to confirm that, when directly compared, reviewers were able to identify a difference in bone healing between the two time points (i.e., the ability to detect subtle change across the 2-week time frame). Categories 4 and 5 were evaluated using the VAS scoring system. Both the craniocaudal and mediolateral projections were utilized in the scoring of the radiographs. Radiographs with slightly altered positioning were shown for both 6-week and 8-week time points. Reviewers were asked to assess bone healing during a single session. Evaluators were blinded by removing all identifying features and labels from the radiographs and labeling them with a random number and letter combination (A-Z and 1–1000).

#### Radiographic scoring

For the radiographic sets comparing recheck radiographs (see Figure 1), reviewers were asked to decide which set appeared more healed (i.e., represents the 8-week timepoint) or whether they are from the same timepoint (i.e., two sets of radiographs representing 8-week timepoint).

For comparison of postoperative radiographs to recheck radiographs the reviewers were asked to provide their subjective assessment of osteotomy healing (i.e., bony bridging, callus formation, osseous remodeling, etc.) of the recheck radiographs based on both radiographic projections (see Figure 4). Reviewers were then asked to move a virtual slider along a 100-point scale. The scale was labeled at either end: 0 with “no healing,” 100 with “completely healed,” with 10% increments marked, and a “pop-up” indicator displaying the precise number selected.

### Data interpretation and analysis

#### Scoring system identification (phase I)

Only sets of radiographs from patients that were correctly interpreted (for the sets that compared 6- and 8-week radiographs) by more than 50% of the reviewers (i.e., confirming a true change in osteotomy healing) were utilized for data comparison.

For sets comparing the postoperative radiograph to a recheck radiograph, the reviewers' scoring of 6- or 8-week radiographs were compared and evaluated for correctness and assigned “correct” (i.e., 8-week osteotomy more healed than 6-week), “incorrect” (i.e., 8-week osteotomy judged less healed than 6-week), and “unchanged” (i.e., 8-week osteotomy judged the exact same amount healed compared to 6-week). For each of the five scoring systems, the proportion of “correct,” “incorrect” and “unchanged” was reported.

#### Effect of positioning (phase II)

For all positioning comparisons of interest, a paired *t*-test and Wilcoxon signed-rank test was conducted. The Shapiro-Wilk test of normality was performed to ensure the data analyzed followed a Gaussian distribution. For comparisons where the test of normality showed p>0.05, the Wilcoxon signed-rank test was used for analysis. For comparisons where the test of normality showed *p* < 0.05, the paired *t*-test was used for analysis.

## Results

### Scoring system identification

Radiographs were evaluated by 10 reviewers (5 board-certified veterinary surgeons, and 5 board-certified veterinary radiologists); 8 reviewers completed all three sessions, 2 reviewers completed one session. A total of 20 radiographic sets (1,352 total responses) from 4 dogs were compared and used for analysis. Radiographs were made of all four dogs at 6-weeks (range: 41–42 days) and at 8-weeks (range: 55–58 days) postoperatively, with an average of 14 days (range: 13–16 days) between the two groups. The 12 comparison sets of recheck radiographs (312 total responses) confirmed a discernible difference in bone healing between 6-weeks and 8-weeks postoperative for all patients (ranging from 78 to 90% correct identification of a change in bone healing). No reviewers were excluded.

For each grading system, there were 208 responses (equally divided between 6- and 8-weeks postoperative). Proportions for each scoring system are provided in Table 1. When using the VAS system, reviewers provided the most correct responses with 76% and the least number of combined unchanged and incorrect responses with 24%. In comparison, using the BRIDGING system, reviewers did not identify any sets incorrectly. However, they also provided the fewest correct responses with only 21% and it had the most combined unchanged and incorrect responses at 79%.

**Table 1:** Responses for 5 different scoring systems comparing healing of TPLO osteotomy.

**RESPONSE**	**VAS**	**DESCRIPTOR**	**SUB11**	**SUB5**	**BRIDGING**
Correct	76%	65%	63%	37%	21%
Incorrect	9%	7%	2%	14%	0%
Unchanged	15%	28%	35%	49%	79%
Unchanged + Incorrect	24%	35%	37%	63%	79%

Responses were judged correct (an increase in healing), incorrect (decrease in healing), or unchanged (same score for both sets). Results were determined from a total of 104 scores from each scoring system.

### Effect of positioning

Radiographs were evaluated by 39 reviewers (32 board-certified veterinary surgeons, 5 board-certified veterinary radiologists, and 2 veterinary surgery residents). A total of 17 radiographic sets (663 total responses) from 5 dogs were compared. Three reviewers were excluded due to 2 or more incorrect responses on the comparison of recheck radiographs, leaving 612 total responses for analysis. The 6 comparison sets of radiographs confirmed the remaining reviewers' ability to identify discernible differences in bone healing between 6- and 8-weeks postoperative for all patients (ranging from 92 to 100% correct identification of a change in bone healing). For all patients in the study, the 6- and 8-week recheck radiographs were taken 41–42 and 55–58 days postoperative.

For all 3 patients the average difference between VAS-scores for all reviewers assessing the same radiographic position at 8-weeks was lower than that from different positions (see Table 2). The magnitude of the difference varied between patients with patient #1 showing the greatest difference. When radiographic sets from 6-and 8-weeks with different positioning were compared for patient #1, the magnitude of change between different positions exceeded the magnitude of comparison of radiographs from the 6- and 8-week time points (see Table 3).

**Table 2:** Influence of positioning of 8-week TPLO-radiographs from the 3 patients evaluated illustrating the different magnitude of changes to the VAS-scoring when reviewers were shown the same radiographic set twice or radiographs positioned differently.

**Patient #**	**1**	**1**	**2**	**2**	**3**	**3**
Number of responses	36	36	36	36	36	36
Comparison sets	D	S	D	S	D	S
VAS-scoring difference in between sets (Average ± SD)	24.53 ± 14.4^*^	3.64 ± 10.8	3.14 ± 4.0^*^	0.33 ± 4.2	3.36 ± 8.0^*^	1.92 ± 5.0^*^
*P*-value	<0.001	0.05	<0.001	0.63	0.02	0.03

D, different position of radiographs.

S, same radiographic set shown twice.

* *P* <0.05.

**Table 3:** Comparison of the influence of positioning and actual healing on osteotomy scoring for patient #1 illustrating that different positioning of TPLO-radiographs results in greater magnitude of changes to the VAS-scoring than the observed amount of healing.

	**Different positioning**		**Different positioning and timepoint**		**Different timepoint**	
Positioning of TPLO radiographs (set A or B)	A/B	A/B	B/A	A/B	A/A	B/B
Time point comparisons (week post-op of set A or B)	6/6	8/8	6/8	6/8	6/8	6/8
Number of responses	36	36	36	36	36	36
Comparison sets	DS	DS	DD	DD	SD	SD
VAS-scoring difference in between sets (Average ± SD)	28.9 ± 16.7^*^	24.5 ± 14.4^*^	36.4 ± 19.0^*^	−17.0 ± 12.7^*^	7.6 ± 9.0^*^	11.9 ± 15.2^*^
*P*-value (paired *t*-test)	<0.001	<0.001	<0.001	<0.001	<0.001	<0.001

DS, Different position of radiographs from the same time point, i.e., illustrating the influence of positioning.

DD, Different position of radiographs from different (6 and 8-week) time point, i.e., illustrating the combined influence of positioning and bone healing.

SD, Same position of radiographs from different (6 and 8-week) time point, i.e., illustrating “true” bone healing.

* *P* < 0.05.

Regardless of the test of normality result, all comparisons shared similar results between the paired *t*-test and the Wilcoxon signed-rank test except for patient #1's 8-week same radiograph comparison. The Shapiro-Wilk test for this comparison was *p* = 0.10, necessitating the use of the paired *t*-test of *p* = 0.05 over the Wilcoxon signed-rank test of *p* = 0.03.

## Discussion

This study was designed to report on the ability of five scoring systems to identify subtle changes in bone healing after TPLO. The goal was to identify a scale that most effectively identifies subtle changes in osteotomy healing after TPLO while limiting incorrect or unchanged scores. As expected, scoring systems utilizing small scales demonstrated a lower rate of incorrect responses but showed a reduced ability to detect small changes. Using the VAS scoring system, reviewers had the highest percentage of correct responses and the lowest percentage of combined unchanged and incorrect responses. Next, the VAS system was utilized to determine whether variability in positioning of TPLO radiographs results in a clinically relevant change of osteotomy healing score. Based on the results of this study, the VAS scoring system may prove to be the system of choice in clinical practice and future research that aims to quantify bone healing after TPLO. This study also indicates the influence of positioning exceeds the measurable bone healing observed over a 2-week period, thus highlighting the importance of consistent/repeatable positioning for radiographs in evaluation of healing and for future studies.

The BRIDGING system had a 0% incorrect response rate, meaning that none of the reviewers falsely identified an increase in healing score. While this seems desirable, the scoring system also had the highest number of unchanged responses. As such, it would be near impossible to detect subtle change in osteotomy healing using this system. Focusing too heavily on minimizing incorrect scores will result in a scoring system that lacks the sensitivity to detect subtle healing, as exemplified by the BRIDGING system. To identify small changes in osteotomy healing, the percent correct criteria is most relevant, as a system that misses a difference will be unable to identify small changes. The binary BRIDGING system, with its narrow assessment criteria, represents one end of the spectrum of scoring systems tested, while the VAS system represents the other end of the spectrum with a broad 0–100 scale. While it might logically be assumed that the VAS system would have the lowest number of unchanged responses due to its large scale, it is unexpected that it also has the highest number of correct responses.

The second-best system (based on the combination of incorrect/unchanged and correct response percentages) was the DESCRIPTOR system, a system that employs clearly defined criteria. The approach of asking directed questions specific to osteotomy healing (e.g., osteotomy margins, caudal osteotomy step) intuitively makes sense and allows one to present more clearly defined criteria; however, these questions only represent one aspect of the TPLO healing. In our study, the radiographic scoring system with the least defined criteria and the broadest scale (the VAS system) scored the most correct responses among reviewers. A logical explanation may be that the less defined scales allow reviewers to use their individual training and criteria for judging TPLO osteotomy healing. It may be, however, that less experienced reviewers may benefit from guided questions as with other potential scoring systems. All reviewers in our study were experienced, as such, we are unable to answer this question. With respect to experience, a broad benefit of a scoring system with fewer defined criteria, in addition to allowing use of individualized training, is the lack of need for specific training to utilize the system.

The VAS system has been designed to apply an objective measurement to a subjective variable that is on a continuum (26). In veterinary medicine, VAS scores have been used for a range of indications, including assessment of lameness (27), nausea (28), pruritus (29), urinary incontinence (30), and pain (31). The VAS has also been used to judge osteoarthritis and for synovitis grading (32, 33). For scoring synovitis severity, the VAS system was found to have low intra-observer variability and moderate inter-observer variability (32). This variability finding is consistent with individual training having an influence on scores and was also represented in our data. VAS is traditionally completed in written form, consists of a 10 cm line where the minimum and maximum scores are positioned at the left and right ends of the line, respectively. The distance from the minimum score (or left end of the line) is measured and allocated as the score. Some scales have modifications, such as marking the midpoint (i.e., 5 cm) to indicate 50% score in the outcome variable(s), such as 50% pain intensity. Similarly, other intervals can be marked on the scale to designate other predetermined scores (29). Comparatively, in our study we used an online VAS where the observer could select a percentage (0–100%) to score the degree of osteotomy healing by using a virtual slider. The slider was marked with 10% increments and indicated the precise number selected. This is similar to the method used by Morgan et al. (30) for assessing urinary incontinence however its validity was not tested. It is unknown whether the method of VAS scoring (i.e., electronic or written) influences assessment. Like the VAS system, the SUB11 system has a large scale but more defined criteria which may result in improved scoring. However, reviewers using this system failed to detect subtle changes 35% of the time.

A fundamental concern when evaluating scoring systems for TPLO osteotomy healing assessment in the clinical setting is the lack of an imaging gold standard. However, true validation requires comparison of a diagnostic test (radiographic scoring of bone healing in this instance) to a recognized gold standard (such as biomechanical and/or histologic assessment of bone healing in this instance). Given the clinical nature of the study, comparison to such a gold standard was not feasible. To utilize radiography as the imaging modality, we addressed this concern by confirming the assumption that the patient's osteotomy in the radiographs taken at 8-weeks is distinguishable from the osteotomy at 6-weeks radiographically due to an increase in healing. This assumption was confirmed *via* the 6-week vs. 8-week comparison sets showing a 78% or greater correct identification rate. While it is recognized that radiographic assessment of bone healing only serves a surrogate measure for osteotomy healing, it remains as the most utilized tool; therefore, pursuit of methods that improve the accuracy of radiographic osteotomy assessment is a step toward optimization of this tool. This approach can be useful for future studies, avoiding the necessity of more invasive methodologies such as biomechanical testing and limiting the expense of advanced imaging.

This study has shown the VAS system to be useful in identifying subtle bone healing by looking at healing over a 2-week interval. In comparison, Walker et al. evaluated the modified radiograph union scale for tibial fractures (mRUST) system compared to the subjective evaluation of radiographic osteotomy union utilizing radiographs taken at an average of 8.4 weeks postoperative (11). The mRUST system is similar to the DESCRIPTOR system in terms of specifically evaluating the cortical margins although it does not allow for the same completeness ratings (24). While the results of the work by Walker et al. suggests that the mRUST system is useful in answering whether the osteotomy is healed, the VAS system has been shown useful at identifying subtle osteotomy healing. As the mRUST system may prove effective in evaluating range of osteotomy healing, further research comparing these two systems' efficacies for subtle osteotomy healing may be indicated.

It should be noted that current recommendations are to place less emphasis on *p*-values from statistical analysis but rather to evaluate the data within the clinical relevance and context (34, 35). Thus while results were interpreted with a *p*-value cut-off in mind, this was not the sole criteria used in determining significance of results.

The circular osteotomy performed with the TPLO in combination with the obscuring imposition of the implants poses a significant challenge for assessment of healing compared to transverse osteotomies or fractures. Identical positioning between rechecks and clear identification of the osteotomy is necessary to avoid artifactual changes in healing scores. In phase II of the study, all three patient's same radiograph comparison had a smaller difference in answers than the different position comparison, indicating positioning made a difference in for the interpretation of all three patients (see Table 2). While reviewers for patients #1 and 2, when shown the same radiograph with slight image adjustments, assessed these radiographs to have healed a similar amount. However, when reshown patient #3's radiograph, it was judged different enough to result in a *p* < 0.05. While many reviewers judged patient #3's same radiographs shown twice to be similar, as evidenced by a mean difference of only 1.92%, there was a relative variation in answers. The significance of this finding is unknown. To test the significance, additional patients should be evaluated. As the remaining two patients' same radiographs shown twice were judged to have healed a similar amount, we conclude that positioning influences the assessment of TPLO osteotomy healing across patients.

Between patients #1 and 2, there is a difference in magnitude when comparing the healing scores and the effect of positioning. The difference in magnitude of these scores may be due to the extent of alteration of the position between patients or individual patient variation. The 8-week postoperative radiographs were obtained in as similar position to the immediately postoperative radiographs. However, the variation in positioning was not standardized and so the magnitude of the effect on the healing score likely varied as a result. Additional testing could be performed to determine the exact type of position alteration that causes the effect on healing score but is likely not necessary for clinical or research purposes. For all radiographs where an evaluation of TPLO osteotomy line healing is required, the focus should be on obtaining similar positioning throughout the healing process for the most accurate assessment.

Patient #1 demonstrates a similar scale of difference for the effect of positioning on TPLO healing scores at two separate time points−6- and 8-weeks postoperative. This suggests that positioning causes a consistent effect on an individual patient's healing scores as that patient heals over time. Further, the effect of positioning appears to be compounded with time. When positioned identically, Patient #1 had an increase in VAS healing score of 7.6–11.9% over 2 weeks. This increase in score represents the “true” amount of bone healing over 2 weeks (i.e., the effect of only time on healing scores). Over this same period, with a slight variation in positioning of the radiographs, healing either decreased by 17% or increased by 36%. This 53% variation in healing scores over a 2-week period demonstrates the profound effect positioning can have when attempting to identify subtle osteotomy healing changes over time. All future studies evaluating TPLO osteotomy healing will need to be precise in their limb positioning to minimize this effect on healing scores.

There were several limitations to this study. To make the study feasible, we chose to use a larger number of reviewers, rather than a larger number of radiographs, patients, and scoring systems which reduces the sample size and limits the variability of the images provided but prevents reviewer fatigue. There were additional TPLO scoring systems considered for this study, including a system that focused on the degree of rounding of the caudal osteotomy margin (9). However, due to the similarities between those systems and the ones tested in this paper, they were not included. Images were carefully chosen to represent common scenarios; however, our results may not be applicable to all TPLO radiographs. Not all reviewers were able to complete all sessions which further reduced sample size. However, this study's focus was on positioning technique while obtaining radiographs and evaluating each radiograph more often, which provides a better evaluation of scoring systems than if these are not prioritized. The small sample size and variation of test results (binary, ordinal, and continuous), negated the possibility of performing meaningful statistical analysis of inter-and intra-observer variability and scoring system results were limited to descriptive analysis.

Given that the VAS score is easy to implement and performed the best in our study setting, it is the authors' choice for future TPLO osteotomy scoring. Additionally, care in positioning of the limb during radiographs should be taken in future TPLO osteotomy healing research.

## Conclusion

The VAS scoring system allowed reviewers to most frequently correctly identify small changes in bone healing in dogs following TPLO. Of the systems examined, the VAS scoring system is a viable, easy to use, and easy to interpret grading system for use in evaluation of how different variables affect TPLO osteotomy healing. In addition, it can be used in the clinical setting to provide a more defined assessment of an individual patient's progress. Positioning of the limb during radiographic imaging following TPLO surgery will affect the healing score assigned to the osteotomy. This effect appears to be consistent across time periods. Therefore, in both clinical and research settings, care should be taken to ensure as similar limb positioning during radiographic imaging as possible to obtain the most accurate assessment of TPLO osteotomy healing.

## Data availability statement

The original contributions presented in the study are included in the article/supplementary material, further inquiries can be directed to the corresponding author.

## Ethics statement

The animal study was reviewed and approved by CSU VTH CRB #2018-177. Written informed consent was obtained from the owners for the participation of their animals in this study.

## Author contributions

RL organized the database, wrote the draft, and collated all subsequent revisions. NL and FD contributed to conception and design of the study. FD acquired the data. FD and RL performed analysis and interpretation. All authors contributed to manuscript revision, read, and approved the submitted version.
